# Pdx1-Cre-driven conditional gene depletion suggests PAK4 as dispensable for mouse pancreas development

**DOI:** 10.1038/s41598-017-07322-5

**Published:** 2017-08-01

**Authors:** Miao Zhao, Parisa Rabieifar, Tânia D. F. Costa, Ting Zhuang, Audrey Minden, Matthias Löhr, Rainer Heuchel, Staffan Strömblad

**Affiliations:** 1 Department of Biosciences and Nutrition, Karolinska Institutet, Stockholm, Sweden; 2 Susan Lehman Cullman Laboratory for Cancer Research, Department of Chemical Biology, Ernest Mario School of Pharmacy, Rutgers, The State University of New Jersey, Piscataway, New Jersey USA; 3 Division of Surgery, CLINTEC, Karolinska Institutet, Stockholm, Sweden; 4 Center for Digestive Diseases, Karolinska University Hospital, Stockholm, Sweden

## Abstract

Constitutive depletion of p21-activated kinase 4 (PAK4) in the mouse causes embryonic lethality associated with heart and brain defects. Given that conventional gene depletion of PAK1 or PAK3 caused functional deficits in the mouse pancreas, while gene depletion of PAK5 or PAK6 did not, we asked if PAK4 might have a functional role in pancreas development. We therefore introduced conditional, Pdx1-Cre-mediated, pancreatic PAK4 gene depletion in the mouse, verified by loss of PAK4 protein expression in the pancreas. PAK4 knock-out (KO) mice were born at Mendelian ratios in both genders. Further, morphological and immunohistochemical examinations and quantifications indicated that exocrine, endocrine and ductal compartments retained the normal proportions and distributions upon PAK4 gene depletion. In addition, body weight records and a glucose tolerance test revealed no differences between WT and PAK4 KO mice. Together, this suggests that PAK4 is dispensable for mouse pancreas development. This will facilitate future use of our Pdx1-Cre-driven conditional PAK4 KO mouse model for testing *in vivo* potential functions of PAK4 in pancreatic disease models such as for pancreatitis and different pancreatic cancer forms.

## Introduction

P21-activited kinases (PAKs), comprising a Rho GTPase-regulated serine/threonine kinase family, have been extensively studied and are implicated in many cellular processes, including cytoskeletal organization, cell cycle and cell survival^1–3^. PAKs are also involved in cancer progression, neuronal diseases, immunity and vascular disorders^1, 3^. While complete gene depletion of PAK2 and PAK4 in mice caused embryonic lethality^4–6^, mice with depletion of PAK1, PAK3, PAK5 or PAK6 remain viable, albeit overt phenotypes^4, 6–12^. PAK1 and PAK3 KO mice display defects in immune, neuronal and metabolic systems^7, 8^, and a combined depletion of the PAK5 and PAK6 genes leads to impairment of mobility, memory and learning^9, 11^. Interestingly, PAKs also play critical roles in the pancreas function, since PAK1 depletion caused deficits in glucose clearance^13^ and PAK3 depletion triggered glucose intolerance under high-fat diet^14^. However, PAK5, PAK6 and PAK5/PAK6 double KO mice display normal pancreas development^9–12^.

PAK4 is highly expressed throughout development as well as in several cancer forms and it is ubiquitously expressed at low levels in many adult tissues^15–17^. PAK4 regulate many important cellular processes, such as cell cytoskeleton dynamics, cell migration, proliferation and survival^17^. PAK4 acts at least in part by exerting its kinase activity towards effector proteins, including LIMK1^18^, paxillin^19^, integrin β5^20^, Ran^21^ and BAD^22^. PAK4 is also essential during development, because PAK4 KO caused embryonic lethality in mouse as a consequence of heart defects and abnormalities in extra-embryonic tissues and the embryonic vasculature^5^. Furthermore, conditional PAK4 KO in the central nervous system triggered growth retardation and premature lethality^23^, while conditional PAK4 KO in the heart provoked abnormal development of the outflow tract and thinning of the wall of the right ventricle^24^. However, the potential role of PAK4 in the pancreas has remained unknown.

To this end, we here generated conditional PAK4 KO mice targeting the pancreas by crossing Pdx1-Cre mice with PAK4^F/F^ mice. Pdx1-Cre-driven PAK4 KO mice were viable and fertile, exhibited normal body weight as well as normal exocrine, endocrine and ductal cell morphology and displayed normal glucose tolerance. These results suggest that PAK4 is not essential for mouse pancreas development. This mouse model may therefore be used to test the potential *in vivo* functions of PAK4 in pancreas disease models, such as for pancreatitis and different pancreas cancer forms.

## Results

### Mice with Pdx1-Cre-driven conditional PAK4 gene depletion are viable

**Figure 1:**
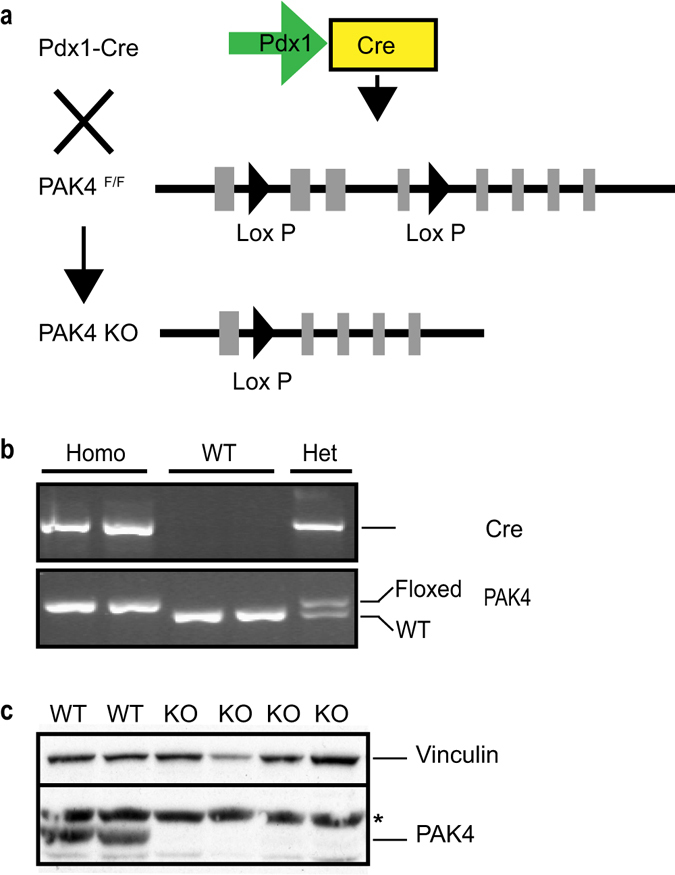
Generation of a mouse model for conditional PAK4 gene depletion in the pancreas. (**a**) Graphical representation of our strategy for generation of a mouse model with conditional PAK4 gene depletion in the pancreas. Exons are indicated by light grey rectangles and Lox P sites are indicated by black triangles. Pdx1-driven Cre expression and consequent recombination of LoxP sites results in the depletion of exons 2–4 in the mouse PAK4 gene as previously described^26^. (**b**) PCR analysis of genomic DNA from 12 days old mouse tails. Upper panel shows the presence of the Pdx1-Cre allele and the lower panel shows PAK4; the upper band in the lower panel displays the floxed PAK4 allele, while the lower band in the lower panel displays a PAK4 WT allele. Thus, appearance of the upper band alone displays homozygous floxed PAK4 allele; the lower band alone represents WT PAK4 allele; while both bands together mean that the mouse is PAK4 heterozygous, i.e. one WT and one floxed allele. Full gels are showed in supplementary Fig. S1a and S1b. (**c**) PAK4 deletion in the mouse pancreas. Immunoblot analysis of pancreatic whole cell lysates shows PAK4 expression in WT pancreas, whereas PAK4 protein expression is below the detection limit in PAK4 homozygous KO mice. Vinculin was used as a loading control. *Shows unspecific bands. Full blot is showed in supplementary Fig. S1c.

To determine the role of PAK4 in pancreatic development, we generated mice with conditional PAK4 depletion in the pancreas epithelial compartments by breeding a Pdx1-Cre mouse strain^25^ with a PAK4^floxed/floxed^ (PAK4^F/F^) strain^26^ (Fig. 1a). Cre and PAK4 were genotyped by PCR analysis of genomic DNA isolated from the mouse tail, identifying wild type (WT) mice, as well as homozygous (Homo) and heterozygous (Het) PAK4 knockout mice (Fig. 1b). When Pdx1-Cre; PAK4^F/+^ mice were crossed with PAK^F/F^, 147 viable offspring were obtained (Table 1). Mice from the four different genotypes were born at the expected Mendelian ratio, i.e. WT (PAK^F/F^ and PAK4^F/+^) 51%; Het (Pdx1-Cre; PAK4^F/+^) 25%; and Homo PAK4 KO (Pdx1-Cre; PAK4^F/F^) 24%. Within the same genotypes, female and male displayed an approximately equal distribution; suggesting that loss of PAK4 does not affect survival in any of the sexes (Table 1). Further, immunoblotting of pancreatic whole cell lysates showed that the PAK4 protein was below the detection limit in PAK4 KO mice, indicating a substantial decrease in PAK4 expression (Fig. 1c). Upon examination of littermates, WT and PAK4 KO groups were similar in size, appearance of the fur, gross morphology and behavior.

**Table 1:** Genotypes of the progeny from Pdx1-Cre; PAK4^F/+^ × PAK4^F/F^.

Total	PAK4^F/F^		PAK4^F/+^		Pdx1-Cre; PAK4^F/+^		Pdx1-Cre; PAK4^F/F^	
147	40 (27%)		35 (24%)		37 (25%)		35 (24%)	
	Female	Male	Female	Male	Female	Male	Female	Male
	20 (50%)	20 (50%)	16 (46%)	19 (54%)	17 (46%)	20 (54%)	17 (48%)	18 (52%)

### Histomorphology of pancreatic tissue upon conditional PAK4 KO resembles that of WT

**Figure 2:**
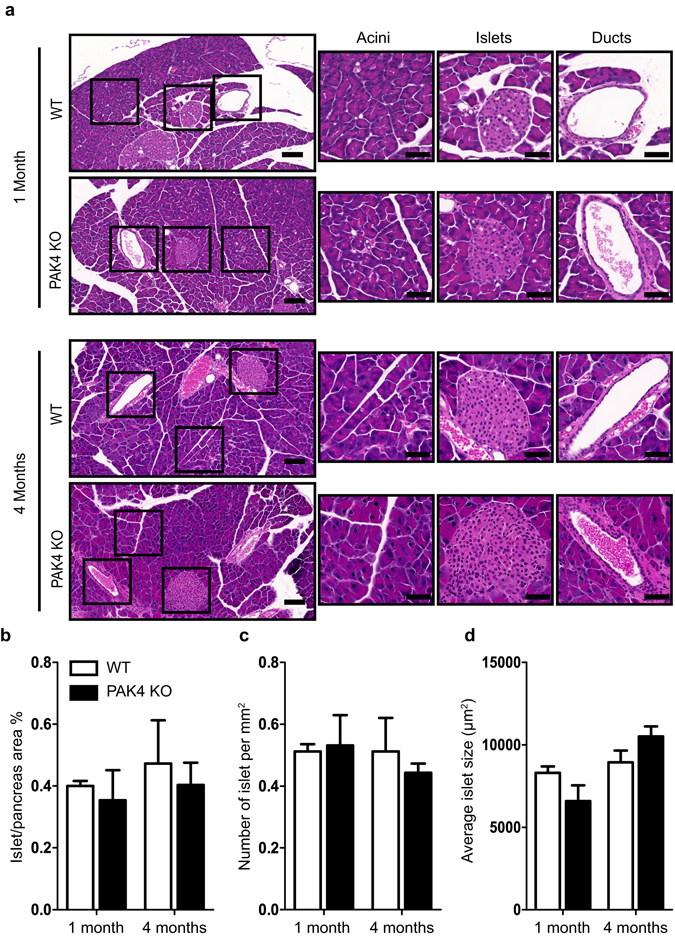
Histomorphology of pancreatic tissue upon conditional PAK4 KO resembles that of WT. (**a**) Representative images of pancreatic sections of WT and PAK4 KO mice of one and four months of age stained with hematoxylin and eosin (H&E). Acini, islets and ducts developed normally in both genotypes. Marked boxes in the left are displayed at higher resolution to the right. Scale bars: 100 μm in the overview image and 50 μm in the inserts (**b**,**c**,**d**) Morphometric data of the H&E-stained pancreatic sections. (**b**) The bars display the percentage of islet area per pancreas, 1 month (p = 0.6046, n = 3–4) and 4 months (p = 0.6527, n = 3–4). (**c**) The number of islets per square millimeter of pancreatic area, 1 month (p = 0.8290, n = 3–4) and 4 months (p = 0.5092, n = 3–4). (**d**) Average islet size. 1 month (p = 0.1205, n = 3–4) and 4 months (p = 0.1573, n = 3–4). Data are expressed as the mean ± SEM. T-test revealed no discernable differences between the genotypes.

To evaluate possible histomorphological alterations in the murine pancreas upon PAK4 depletion, paraffin embedded pancreatic sections from mice of one and four months of age were primarily stained with hematoxylin and eosin (H&E). These stainings revealed that the acinar structures in PAK4 KO pancreases were evenly distributed and resembled those in WT mice (Fig. 2a). Of note, the eosin staining in the acinar cell cytoplasm showed equal in strength (Fig. 2a), suggesting that the amount of zymogen granules were similar in WT and PAK4 KO mice. No obvious difference in ductal structures between the cohorts was observed at one and four months of age (Fig. 2a). Further, islets were scattered randomly between the acini in both groups (Fig. 2a).

To explore potential subtle differences in islets of Langerhans between WT and PAK4 KO mice, we measured the area fraction covered by islets in entire pancreatic sections (Fig. 2b), and quantified the number of islets per pancreatic area (Fig. 2c). The obtained results were similar for both genotypes. Consistent with previous observations^27^, the average islet size increased with age, but no significant differences were detected between WT and PAK4 KO mice (Fig. 2d).

### Normal occurrence of the various cell types in exocrine and endocrine pancreas upon conditional PAK4 depletion

**Figure 3:**
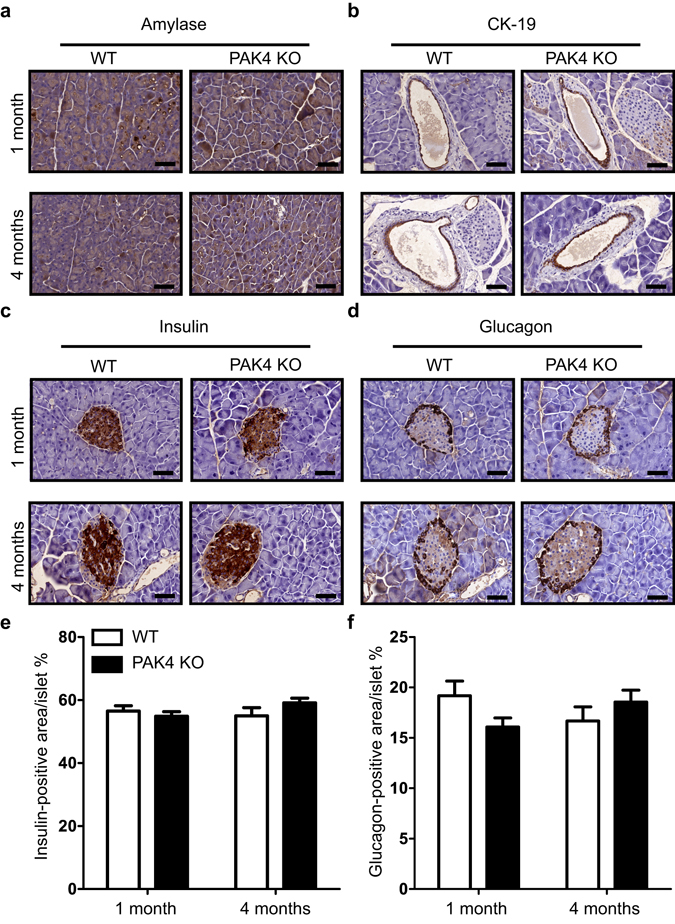
Normal occurrence of the various cell types in exocrine and endocrine pancreas upon conditional PAK4 depletion. (**a**,**b**) Immunohistochemical staining for the exocrine markers amylase **(a)** and CK-19 (**b**) in pancreas from WT and PAK KO mice of one and four months of age. Scale bars: 60 μm **(c-d)** Two adjacent pancreatic sections from 1 and 4 months old WT and PAK KO mouse pancreas were stained with anti-insulin (**c**) and anti-glucagon (**d**) antibodies, respectively. Scale bars: 40 μm (**e**) Morphometric data of the Insulin-stained islet area. Data are expressed as the mean ± SEM. T-test revealed no discernible differences between the genotypes. 1 month (p = 0.4706, n = 3) and 4 months (p = 0.1525, n = 3–4). (**f**) Morphometric data of the Glucagon-stained islet areas. Data are showed as the mean ± SEM. T-test revealed no discernible differences between the genotypes. 1 month (p = 0.1094, n = 3–4) and 4 months (p = 0.3168, n = 3).

The localization and proportion of different cell types are critical for the pancreas to execute its functions^28^. To compare exocrine, endocrine, and ductal structures, sections from paraffin embedded pancreases were labelled for the exocrine markers amylase and CK-19 and the endocrine markers insulin and glucagon. Amylase staining showed similar expression level in acini throughout the entire pancreas (Fig. 3a), while CK19 stained ductal structures revealed a branched ducts system in the whole pancreas (Fig. 3b) with no differences between WT and PAK4 KO mice at neither one or four months of age. Also, the endocrine markers labeled islets in the expected pattern in both groups of mice. Insulin staining β-cells formed the majority of the islets (Fig. 3c), while glucagon staining α-cells localized at the periphery of the islets (Fig. 3d). We next performed quantitative analyses of the islets by staining two adjacent sections with anti-insulin and anti-glucagon antibodies. However, no significant differences were observed between WT and PAK4 KO mice (Fig. 3e,f). In summary, expression of exocrine and endocrine markers in PAK4 KO pancreases appears equivalent to that of WT.

### PAK4 KO mice have normal body weight and glucose regulatory function

**Figure 4:**
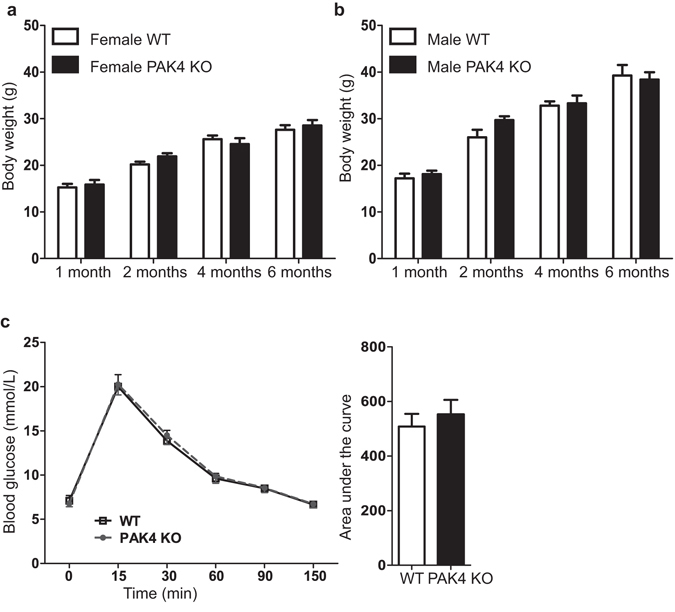
PAK4 KO mice have normal body weight and glucose regulatory function. (**a**) The body weight of 1, 2, 4, and 6 months old mice were from (**a**) female and (**b**) male WT and PAK4 KO groups. Results are means ± SEM. T-test revealed no discernible differences between the genotypes. 1 month-female (p = 0.5618, n = 4); 2 months-female (p = 0.0864, n = 12 (WT) and 6 (PAK4 KO)); 4 months-female (p = 0.4756, n = 9(WT) and 6 (PAK4 KO)); 6 months-female (p = 0.5589, n = 11 (WT) and 16 (PAK4 KO)); 1 month- male (p = 0.4834, n = 14); 2 months-male (p = 0.0880, n = 5–6); 4 months-male (p = 0.7804, n = 12 (WT) and 7 (PAK4 KO)) and 6 months-male (p = 0.6544, n = 4). (**c**) Glucose tolerance test in WT and PAK4 KO mice. Following 6 h of fasting, 8 weeks old female mice were injected intraperitoneally (IP) with 2 g/kg glucose. Blood glucose was measured at the indicated time points after intraperitoneal glucose injections. Data are expressed as the mean + SEM. The left graph shows the time. The right bar graph displays the area under the time curve. Data are expressed as the mean ± SEM. T-test revealed no discernible differences between the genotypes (p = 0.5428, n = 8).

Insulin and glucagon are important hormones for the regulation of the body weight^29, 30^. We therefore examined whether conditional PAK4 gene depletion may affect the body weight at different ages. Analysis of both female and male cohorts at one, two, four and six months of age showed an expected increase in body weight with age, but with no discernible differences between the two genotypes (Fig. 4a and b).

Maintaining blood glucose homeostasis is one of the important roles of the endocrine pancreas^31^. To evaluate whether conditional PAK4 gene depletion may affect this pancreatic function, we performed a glucose tolerance test using intraperitoneal (IP) glucose injections in mice at 2 months of age. As shown in Fig. 4c, the plasma glucose concentration peaked at 15 min after the glucose challenge and then gradually returned to normal level throughout the experiment. Importantly, WT and PAK4 KO mice displayed identical glucose induction and clearance curves, indicating that PAK4 gene depletion in the pancreas does not affect the glucose regulatory function.

## Discussion

PAK4 is highly expressed throughout development and ubiquitously expressed at low levels in many adult tissues^5^. Constitutive PAK4 depletion causes embryonic lethality^5^ and although mice with conditional PAK4 gene depletion in the heart and in the central nervous system are viable, they displayed serious organ defects^23, 24^. Given that PAK4 is ubiquitously expressed, including in the pancreas, we generated a mouse model with conditional genetic depletion of PAK4 in the pancreatic compartments, but found no obvious alterations on pancreas development and function. This suggests that PAK4 is dispensable for murine pancreatic development and function. It is possible that pancreatic function may instead rely on other PAK family members, since genetic depletion of PAK1 caused a defect in glucose homeostasis, inefficient insulin secretion, and abnormal glucose clearance^13^ and PAK3 deletion affected glucose tolerance upon exposure to high fat diet^14^. Interestingly, genetic depletion of the other PAK family members PAK5 and PAK6, as well as PAK5/PAK6 double gene KO, all resulted in normal pancreas development^9–12^. This supports the notion that the different PAK family members fulfill distinct functions. However, we cannot rule out that PAK4 may play a role in the pancreas upon active challenge, since we have not examined the potential function of PAK4 under challenged conditions.

Importantly, the pancreas is sensitive to different prevalent diseases, such as pancreatitis^32, 33^, type 1 diabetes^34^, neuroendocrine tumors and pancreatic adenocarcinoma^35^. Among these, PAK4 has been found amplified and overexpressed in pancreatic adenocarcinoma cell lines as well as in cancer patients with this disease^2, 36–42^. However, our mechanistical understanding of these diseases remains limited. Given that complete PAK4 gene depletion in the mouse causes embryonic lethality, our model for conditional PAK4 gene depletion in the mouse pancreas will become a useful tool to examine the role of PAK4 in diseases of the pancreas. This usefulness of our model is further enhanced by the fact that PAK4 gene depletion in the pancreas in this model caused no apparent pancreas developmental defects that may otherwise interfere with the disease models.

In conclusion, PAK4 appears to be dispensable for mouse pancreas development and therefore, our mouse model of conditional PAK4 gene depletion in the pancreas can be useful for testing potential *in vivo* functions of PAK4 in pancreatic diseases, such as pancreatitis and different forms of pancreatic cancer.

## Methods

All methods were performed in accordance with relevant guidelines and regulations.

### Mouse Strains

Pdx1-Cre mice and PAK4^F/F^ mice were generated as previously described^25, 26^ and these PAK4^F/F^ mice were previously used to identify critical roles of PAK4 in the development of the heart and central nervous system^23, 24^. For conditional gene deletion in the pancreas, PAK4^F/F^ mice were first crossed with Pdx1-Cre mice to generate Pdx1-Cre; PAK4^F/+^. Such animals were then crossed with PAK4^F/F^ mice, resulting in littermates with PAK^F/F^, PAK4^F/+^, Pdx1-Cre; PAK4^F/+^ and Pdx1-Cre; PAK4^F/F^ genotypes (Table 1). The mice had access to food and water ad libitum, 12 h light/dark cycle, controlled humidity (55% ± 5%) and temperature (21 °C ± 2 °C). All animal experiments were approved by the Stockholm South Animal Ethics Committee and were performed in accordance with Karolinska institutet’s guidelines and animal welfare regulations of Sweden.

### Tissue collection

Mice were killed by cervical dislocation and the pancreas was collected. For protein extraction, the pancreas was snap-frozen in liquid nitrogen and stored at −80 °C. For immunohistochemistry, the pancreas was fixed with 4% paraformaldehyde (P6148, Sigma) overnight, and then washed with phosphate-buffered saline (PBS) and kept in 70% ethanol for paraffin embedding.

### Genotyping by PCR

Genomic DNA was extracted from 12–19 day old mouse tails using the fast tissue–to-PCR kit (#K1091, Fermentas). Primers were synthesized by Thermo Fisher Scientific. The genotyping primer sequences were as follows: Pdx1-Cre-F: AACATGCTTCATCGTCGG; Pdx1-Cre-R: TTGCCCCTGTTTCACTATCCAG; PAK4-F: CGGATATTGTCACCCACACCAG; PAK4-R: CTAACAGGGACAGGAGCT. The PCR was performed in 20 μL reaction volume containing 10 μL tissue green PCR master mix (2X) (K1082, Thermo Fisher Scientific), 0.5 μM forward and reverse primers, 4 μL tissue extract, 4 μL ddH_2_O. The PCR reaction was carried out in a thermal cycler as follows: initial denaturation for 2 min at 95 °C, followed by 30 cycles of denaturation for 1 min at 95 °C, annealing for 30 s (Pdx1-Cre allele at 57 °C and PAK4^F/F^ allele at 62 °C), extension for 1 min at 72 °C, and a final extension of 10 min at 72 °C. The samples were then cooled to 4 °C. Genotypes were visualized by 2.5% agar gel stained with gel red (41003, Biotium).

### Immunoblotting

Tissues were homogenized in RIPA buffer containing Protease (1697498, Roche) and Phosphatase inhibitors (P0044, Sigma) by using a Homogenizer (10768992, Thermo Fisher Scientific). 100 μg of protein samples were separated on SDS-polyacrylamide gels and transferred to an Immobilon-P Membrane (IPVH00010, Millipore). After blocking for 1 h at room temperature with 5% no-fat milk (A0830, Applichem), the membranes were incubated with primary antibodies overnight at 4 °C. Rabbit polyclonal anti-PAK4 antibodies were generated in our laboratory^43^, mouse vinculin antibody (ab11194) was purchased from Abcam. The membranes were then incubated with a horseradish peroxidase-conjugated secondary antibody (Jackson ImmunoResearch) for 1 h at room temperature. Membranes were developed by enhanced chemiluminescence (32106, Thermo Science).

### H&E staining and immunohistochemistry

Fixed mouse pancreases were routinely processed and paraffin embedded. The paraffin-embedded tissues were sectioned at 4 μm. The tissue sections were de-paraffinized in xylene and re-hydrated through a graded series of ethanol. For immunohistochemistry, heat-induced antigen retrieval was performed for 20 min in 10 mM sodium-citrate buffer (pH 6.0) by incubating the slides in a microwave oven. Subsequently, the slides were washed in PBS and incubated for 20 min in 3% hydrogen peroxide in water to block endogenous peroxidase activity. The sections were then washed in PBS and blocked in 1% bovine serum albumin (BSA) with 0.1% NP40 for 1 h in RT. The tissue sections were incubated with the primary antibody in PBS containing 1% BSA and 0.1% NP40 for overnight at 4 °C. Rabbit polyclonal anti-amylase was purchased from Sigma (A8273), rat monoclonal anti-CK19 (TROMA-III) from DSHB, and rabbit polyclonal anti-glucagon (A0565) and guinea pig polyclonal anti-insulin (A0564) from Dako. Subsequently, the sections were washed in PBS and incubated with biotinylated secondary antibodies or HRP secondary antibodies corresponding to the host organism of the secondary antibody in PBS supplemented with 1% BSA and 0.1% NP40. The slides were washed in PBS, incubated with streptavidin peroxidase (50209Z, Life Technologies) for 20 min and washed again in PBS before visualizing the samples using the DAB (K3467, Dako) substrate. The sections were counterstained with hematoxylin (01820, Histolab). All the H&E and Immunohistochemistry slides were scanned using the Pannaromic MIDI II from 3DHISTECH.

### Morphometry

Stained sections were imaged using a Pannaromic MIDI II digital slide scanner and morphometrical parameters were examined as follows. For each animal, three H&E-stained sections were obtained at 100-µm intervals (n = 3–4 for 1-month-old mice, and n = 3–4 for 4-month-old mice). The total islet number, the size of each islet, and the pancreatic area were counted or analyzed using the software CaseViewer from 3DHISTECH. Briefly, in each section from WT and PAK4 KO groups, islet/pancreas area was the total islet area in a section divided by the total pancreas area of that section; the number of islet per mm^2^ pancreas area was determined by dividing total islet number by total pancreatic area (mm^2^). The average islet size was determined by averaging the size of each islet per section. In sections stained with anti-insulin or anti-glucagon antibodies (n = 3–4 for 1-month-old mice, and n = 3–4 for 4-month-old mice), the positive area and the size of each islet were measured using Image J software (version 1.48).

### Glucose tolerance test

8 WT and 8 PAK4 KO female mice of two months of age were starved for 6 h before the experiment. Mice were weighed and 2 g/kg body weight sterile glucose (15023021, Thermo Fisher Scientific) was administered via an intraperitoneal (IP) injection with a sterile U300 insulin syringe (230–4533, BD Medical). A small nick at the tail was made on the tail vein with a sterile razor blade, and then a drop of blood was placed onto a test strip and measured by a glucometer (ACCU-CHEK, Roche) at 0, 15, 30, 60, 90 and 150 min.

### Statistics

Graphpad Prism 5 software was used to perform statistical analysis. Values are presented as mean ± SEM. P-values were determined using a two-tail unpaired t-test. P < 0.05 was used as a threshold for statistical discernibility.

### Data Availability

The datasets generated during the current study are available from the corresponding author upon reasonable request.

## Electronic supplementary material

Supplementary information
