# Supplementary material for: Pdx1-Cre-driven conditional gene depletion suggests PAK4 as dispensable for mouse pancreas development
Source: Sci Rep. 2017 Aug 1;7:7031. doi: 10.1038/s41598-017-07322-5 (PMC5539201; doi:10.1038/s41598-017-07322-5)
Supplement: Supplementary file 1 — Supplementary information [file 41598_2017_7322_MOESM1_ESM.pdf]

Supplementary Information

Pdx1-Cre-driven conditional gene depletion suggests PAK4 as dispensable for mouse pancreas development

Miao Zhao, Parisa Rabieifar, Tânia D. F. Costa, Ting Zhuang, Audrey Minden,Matthias Löhr, Rainer Heuchel, Staffan Strömblad

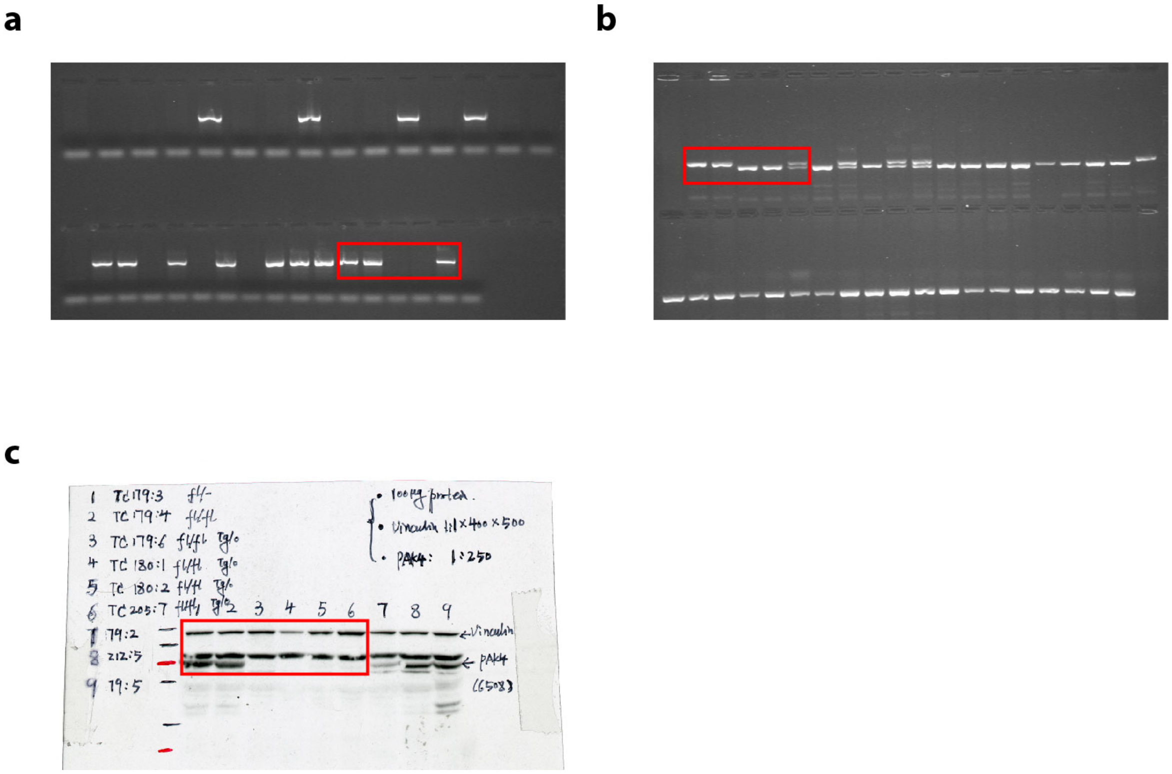

Supplementary Figure S1 Uncropped images for all gels and Western blots.
